# Temporary Telemedicine Policy and Chronic Disease Management in South Korea: Retrospective Analysis Using National Claims Data

**DOI:** 10.2196/59138

**Published:** 2024-11-20

**Authors:** Ji Ye Kang, Weon Jung, Hyun Ji Kim, Ji Hyun An, Hee Yoon, Taerim Kim, Hansol Chang, Sung Yeon Hwang, Jong Eun Park, Gun Tak Lee, Won Chul Cha, Sejin Heo, Se Uk Lee

**Affiliations:** 1 Department of Emergency Medicine Sungkyunkwan University School of Medicine Samsung Medical Center Seoul Republic of Korea; 2 Department of Psychiatry Sungkyunkwan University School of Medicine Samsung Medical Center Seoul Republic of Korea

**Keywords:** telemedicine, public health, medication adherence, COVID-19, chronic diseases

## Abstract

**Background:**

Since its introduction, telemedicine for patients with chronic diseases has been studied in various clinical settings. However, there is limited evidence of the effectiveness and medical safety of the nationwide adoption of telemedicine.

**Objective:**

This study aimed to analyze the effects of telemedicine on chronic diseases during the COVID-19 pandemic under a temporary telemedicine policy in South Korea using national claims data.

**Methods:**

Health insurance claims data were extracted over 2 years: 1 year before (from February 24, 2019, to February 23, 2020) and 1 year after the policy was implemented (from February 24, 2020, to February 23, 2021). We included all patients who used telemedicine at least once in the first year after the policy was implemented and compared them with a control group of patients who never used telemedicine. The comparison focused on health care use; the medication possession ratio (MPR); and admission rates to general wards (GWs), emergency departments (EDs), and intensive care units (ICUs) using difference-in-differences analysis. A total of 4 chronic diseases were targeted: hypertension, diabetes mellitus (DM), chronic obstructive pulmonary disease (COPD), and common mental disorders.

**Results:**

A total of 1,773,454 patients with hypertension; 795,869 patients with DM; 37,460 patients with COPD; and 167,084 patients with common mental disorders were analyzed in this study. Patients diagnosed with hypertension or DM showed increased MPRs without an increase in GW, ED, or ICU admission rates during the policy year. Moreover, patients in the DM group who did not use telemedicine had higher rates of ED, GW, and ICU admissions, and patients in the hypertension group had higher rates of GW or ICU admissions after 1 year of policy implementation. This trend was not evident in COPD and common mental disorders.

**Conclusions:**

The temporary telemedicine policy was effective in increasing medication adherence and reducing admission rates for patients with hypertension and DM; however, the efficacy of the policy was limited for patients with COPD and common mental disorders. Future studies are required to demonstrate the long-term effects of telemedicine policies with various outcome measures reflecting disease characteristics.

## Introduction

Telemedicine is a broad term that includes a wide range of methods used to communicate with patients using technologies, including multiple subtypes, such as telemonitoring, tele-education, teleconsultation, and telecare [1-4]. Since its introduction, telemedicine has been extensively studied in various clinical settings, especially for patients with chronic diseases who need continuous management [3,5-9]. Many reports have provided evidence that telemedicine improves the clinical outcomes of patients with chronic diseases, such as decreasing blood pressure (BP) in patients with hypertension and hemoglobin A_1c_ levels in patients with diabetes mellitus (DM), and reduces the hospitalization rate and improves medication adherence, cost-effectiveness, and quality of life for patients with various chronic diseases, such as chronic obstructive pulmonary disease (COPD) and psychiatric diseases [5,6,10-14].

The COVID-19 pandemic has led to a significant reduction in in-person medical visits globally [15,16]. To address this issue, telemedicine has been widely used in outpatient clinical settings worldwide during the COVID-19 pandemic [17-20]. A meta-analysis review on DM in primary care showed that telemedicine interventions significantly improved glycated hemoglobin levels at 6 months [21]. Various medical devices, such as portable spirometers, video calls, and telephones were used for diagnosis, self-monitoring BP, and follow-up consultation in outpatient clinics [22,23]. Moreover, telemedicine has been used not only for screening suspected COVID-19 cases but also for managing non–COVID-19 cases, primarily in the fields of internal medicine, oncology, and surgery with their chronic complications [24-26].

In South Korea, only a few pilot studies on telemedicine have been conducted, and these studies were limited to a small number of participants in a few isolated regions for a short period [27]. With the outbreak of the COVID-19 pandemic, the health authorities of South Korea permitted temporary teleconsultations and teleprescriptions nationwide for the first time to ensure continuity of care, especially for patients with chronic disease, and to overcome the limited accessibility of medical institutions [28]. However, the introduction of telemedicine requires additional research due to insufficient evidence on medical safety and effectiveness given the national context, making it difficult to assess its impact.

This study aimed to analyze health care use, prescription adherence, and hospitalization under a temporary telemedicine policy during the COVID-19 pandemic, focusing on patients with common chronic diseases to provide evidence for the potential benefits and considerations of introducing telemedicine using national claims data.

## Methods

### Data Source and Study Population

This was a retrospective observational study that followed the STROBE (Strengthening the Reporting of Observational Studies in Epidemiology) guidelines. We used the National Health Insurance Service (NHIS) database of South Korea (NHIS-2022-1-330) [29]. The NHIS is a universal social insurance program that covers 97% of the entire Korean population. The NHIS also manages administrative processes for Medicaid beneficiaries (3% of the population with the lowest income) and reimburses medical professionals for their services. The NHIS database includes information on demographic characteristics, health care use, and diagnostic codes defined by the *Korean Classification of Diseases, 7th Revision* (*KCD-7*), and the *International Classification of Diseases, 10th revision*.

Due to the COVID-19 pandemic, the Korean government temporarily allowed telemedicine in the form of teleconsultations or teleprescriptions beginning on February 24, 2020. Health insurance claims data were extracted for a total of 2 years, 1 year before the policy was implemented (from February 24, 2019, to February 23, 2020) and 1 year after the policy was implemented (from February 24, 2020, to February 23, 2021).

We included all patients who used telemedicine at least once 1 year after the policy was implemented. Telemedicine use was identified with the keywords representing telemedicine in the classification code JX999 (other details) in the database, for which claims are required. However, the code does not specify the modalities of telemedicine. Due to NHIS policy restrictions, we did not receive data for the entire population. Instead, we were provided with a control group at a 5:1 ratio to the telemedicine group, based on age and sex for all telemedicine users. After receiving the data, we defined disease groups using an operational definition tailored to the study’s objectives and extracted the relevant patients accordingly. We excluded individuals who died before the policy was implemented and whose claims data were unavailable to identify outpatient visits or telemedicine through a specification code.

### Setup of Chronic Disease Groups

We included 4 chronic disease groups—hypertension, DM, COPD, and common mental disorder groups—for which telemedicine is frequently used [30-33]. We defined patients with chronic disease as those who were prescribed the relevant drug at least 2 times for the primary or secondary diagnosis 1 year before the implementation of the telemedicine policy. All the diagnosis codes for hypertension (I10-I15) and DM (E10-E14) and all the emphysema and COPD codes (J43-J44) were included in the definitions. For common mental disorders, patients with a diagnosis of schizophrenia and psychotic disease and bipolar, depressive, and anxiety disorders were included (F2x, F31-33, and F41). The major classification categories of prescribed drugs were referred to identify proper relevant drugs for each disease (Multimedia Appendix 1).

### Sociodemographic Variables and Outcome Measures

Sociodemographic variables included the Charlson Comorbidity Index (CCI), residence location, type of disability, and degree of disability [34]. Residence location was categorized into metropolitan cities (cities with more than 1 million people), cities, and rural areas. The detailed definitions of the factors are presented in Multimedia Appendix 2.

We evaluated the health care use rate for each chronic disease group regarding policy implementation. In addition, we evaluated the medication possession ratio (MPR) as a result index to analyze prescription adherence in each disease group [35,36]. The MPR was calculated by dividing the total number of days the medication was prescribed by the number of days in the study period [37]. This study compared the MPR of telemedicine and control groups before and after policy implementation, calculating the MPR for each specific drug based on the corresponding disease. For cases involving multiple drugs, the longest prescription period was used, with duplicate or overlapping days excluded, along with patients who had a history of hospitalization during the analysis period. We evaluated the relevance of each disease and its complications regarding admission to general wards (GWs), emergency departments (EDs), and intensive care units (ICUs) in the telemedicine and control groups after the policy was implemented [38]. Moreover, to overcome the weak relationship between the 1-year admission rate and each type of telemedicine use, we additionally analyzed the rates of admission to GWs, EDs, and ICUs within 1 month after each health care use for both in-person visits and telemedicine visits [39].

### Statistical Analysis

All continuous and categorical variables are reported as the mean (SD) and number (percentage), respectively. To test the differences in categorical variables, the chi-square test or Fisher exact test, when appropriate, was used. The effects of the telemedicine policy on the health care use rate, the MPR, and hospital admission rates were analyzed using difference-in-differences (DID) analysis. DID analysis is a method of confirming the difference in average performance between a treatment group and a control group before and after an intervention and was mainly used to confirm the effectiveness of the policy [40]. The DID model specification was as follows:

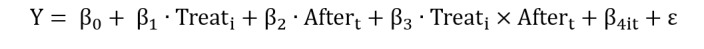



The dependent variable Y corresponds to patient outcome measures, such as health care use, the MPR, and admission rates. The main variable of interest, Treat_i_, indicates whether a patient used telemedicine (control group vs telemedicine group). β_1_ is the DID coefficient that captures the telemedicine use rate after the temporary telemedicine policy was implemented. After_t_ refers to the period after the policy was implemented. Treat_i_ × After_t_ refers to the effect of telemedicine that we aimed to estimate through DID analysis. We include unit-fixed effects, such as age, gender, CCI, disability status, and region.

A *P* value <.05 was considered to indicate statistical significance. Data analysis was conducted in a limited space in the data center of the NHIS Corporation, and the analysis was conducted using R software (version 3.5.2; R Development Core Team) as an analysis tool.

### Ethical Considerations

The use of the NHIS’s customized research database in this study was approved by the NHIS’s Ethics Committee (NHIS-2022-1-330). In addition, the study was approved by Samsung Medical Center’s institutional review board (SMC 2022-01-058). The need for informed consent was waived owing to the observational nature of the study.

## Results

### The Result of the Study Population

**Figure 1:**
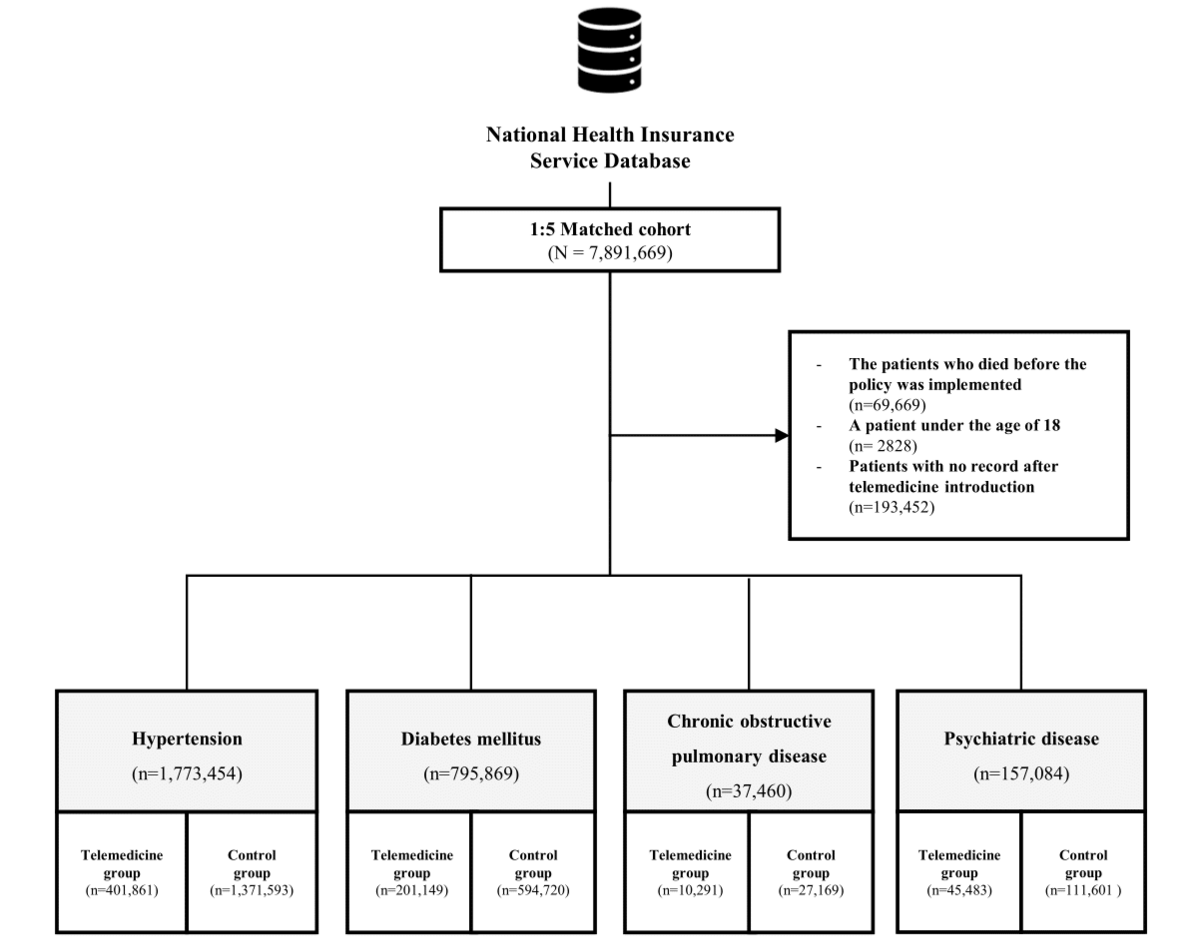
Flowchart of the study population.

A total of 7,891,669 patients, consisting of 1,315,284 patients in the telemedicine group and 6,576,385 patients in the 5-fold matched control group, were initially enrolled by reviewing health insurance claims data. Among these patients, 69,669 patients who died before the telemedicine policy was implemented; 2828 patients who were aged under 18 years; and 193,452 patients who had no claims data after the telemedicine policy was implemented were excluded. According to the operational definitions of the 4 chronic diseases, 1,773,454 patients in the hypertension group; 795,869 patients in the DM group; 37,460 patients in the COPD group; and 167,084 patients in the common mental disorders group were ultimately analyzed (Figure 1).

### Baseline Characteristics of the Study Population

Among the patients in the telemedicine group, more than half were aged 60 years or older in all 4 disease groups (hypertension: 269,973/401,861, 67.2%; DM: 132,678/201,149, 66%; COPD: 9531/10,291, 92.7%; and common mental disorder group: 29,148/45,483, 64.1%). Telemedicine was used more by female patients in hypertension (220,025/401,861, 54.8%) and common mental disorder (31,136/45,483, 68.5%) groups but less by female patients in the DM (97,650/201,149, 48.5%) and COPD (2557/10,291, 24.8%) groups. Approximately half of the patients in the telemedicine group lived in metropolitan cities (hypertension group: 204,092/401,861, 50.8%; DM group: 101,841/201,149, 50.6%; and common mental disorders group: 22,869/45,483, 50.3%), except patients in the COPD group (4369/10,291, 42.5%), and approximately 10% of patients lived in rural areas (hypertension group: 34,412/401,861, 8.6%; DM group: 17,824/201,149, 8.9%; common mental disorder group: 4402/45,483, 9.7%; and COPD group: 1422/10,291: 13.8%). Approximately half of the patients with DM and COPD had high morbidity severity scores, with CCIs greater than 3 points (DM group: 84,164/201,149, 41.8%; COPD group: 4415/10,291, 42.9%), while 25.2% (11,451/45,483) of patients with the common mental disorder and 18.3% (73,459/401,861) of patients with hypertension had CCIs greater than 3 points. Almost all the patients with hypertension, DM, and common mental disorders in the telemedicine group had no disabilities (hypertension group: 349,811/401,861, 87%; DM group: 171,563/201,149, 85.3%; and common mental disorder group: 37,007/45,483, 81.4%); however, the patients with COPD (1241/10,291, 12.1%) in the telemedicine group had the most severe disabilities among the 4 disease groups (Table 1).

**Table 1:** Demographics of participants.

Variables		Hypertension (n=1,773,454)					Diabetes mellitus (n=795,869)						Chronic obstructive pulmonary disease (n=37,460)						Psychiatric disease (n=157,084)				
		Telemed-icine (n= 401,861)	Control (n= 1,371,593)	SMD^a^		Telemed-icine (n= 201,149)		Control (n= 594,720)		SMD		Telemed-icine (n= 10,291)		Control (n= 27,169)		SMD		Telemed-icine (n= 45,483)		Control (n= 111,601)		SMD	
**Age group (years), n (%)**					0.265						0.275						0.068						0.214
	18-39	7179 (1.8)	9227 (0.7)			5402 (2.7)		6043 (1)				28 (0.3)		37 (0.1)				4870 (10.7)		7095 (6.4)			
	40-49	34,710 (8.6)	62,943 (4.6)			18,181 (9)		28,563 (4.8)				117 (1.1)		192 (0.7)				4664 (10.3)		8516 (7.6)			
	50-59	89,999 (22.4)	234,263 (17.1)			44,888 (22.3)		102,860 (17.3)				615 (6)		1404 (5.2)				6801 (15)		14,930 (13.4)			
	60-69	114,618 (28.5)	410,705 (29.9)			59,051 (29.4)		186,448 (31.4)				2373 (23.1)		6159 (22.7)				8983 (19.8)		22,606 (20.3)			
	70-79	88,349 (22)	380,504 (27.7)			45,850 (22.8)		169,441 (28.5)				3931 (38.2)		10,643 (39.2)				10,139 (22.3)		28,654 (25.7)			
	>80	67,006 (16.7)	273,951 (20)			27,777 (13.8)		101,365 (17)				3227 (31.4)		8734 (32.1)				10,026 (22)		29,800 (26.7)			
**Sex, n (%)**					0.072						0.072						0.014						0.044
	Female	220,025 (54.8)	800,174 (58.3)			97,650 (48.5)		310,008 (52.1)				2557 (24.8)		6583 (24.2)				31,136 (68.5)		78,684 (70.5)			
	Male	181,836 (45.2)	571,419 (41.7)			103,499 (51.5)		284,712 (47.9)				7734 (75.2)		20,586 (75.8)				14,347 (31.5)		32,917 (29.5)			
**Residence, n (%)**					0.086						0.066						0.085						0.081
	Metropolis	204,092 (50.8)	663,077 (48.3)			101,841 (50.6)		290,087 (48.8)				4369 (42.5)		11,650 (42.9)				22,869 (50.3)		53,751 (48.2)			
	City	163,357 (40.7)	557,511 (40.6)			81,484 (40.5)		240,701 (40.5)				4500 (43.7)		11,028 (40.6)				18,212 (40)		44,277 (39.7)			
	Rural	34,412 (8.6)	151,005 (11)			17,824 (8.9)		63,932 (10.7)				1422 (13.8)		4491 (16.5)				4402 (9.7)		13,573 (12.2)			
**CCI^b^, n (%)**					0.103						0.058						0.122						0.082
	0	124,454 (31)	474,477 (34.6)			61 (0)		322 (0.1)				0 (0)		0 (0)				12,029 (26.4)		32,305 (28.9)			
	1	129,039 (32.1)	449,586 (32.8)			63,102 (31.4)		200,099 (33.6)				3147 (30.6)		9743 (35.9)				12,737 (28)		32,494 (29.1)			
	2	74,909 (18.6)	238,938 (17.4)			53,822 (26.8)		160,484 (27)				2729 (26.5)		7152 (26.3)				9266 (20.4)		22,048 (19.8)			
	3+	73,459 (18.3)	208,592 (15.2)			84,164 (41.8)		233,815 (39.3)				4415 (42.9)		10,274 (37.8)				11,451 (25.2)		24,754 (22.2)			
**Type of disability, n (%)**					0.022						0.029						0.064						0.056
	Normal	349,811 (87)	1,197,143 (87.3)			171,563 (85.3)		507,598 (85.4)				7419 (72.1)		20,312 (74.8)				37,007 (81.4)		92,180 (82.6)			
	Physical disability	50,323 (12.5)	170,339 (12.4)			27,920 (13.9)		83,628 (14.1)				2817 (27.4)		6761 (24.9)				7076 (15.6)		16,965 (15.2)			
	Psychiatric disability	1727 (0.4)	4111 (0.3)			1666 (0.8)		3494 (0.6)				55 (0.5)		96 (0.4)				1400 (3.1)		2456 (2.2)			
**Degree of disability, n (%)**					0.026						0.027												0.056
	Normal	349,811 (87.0)	1,197,143 (87.3)			171,563 (85.3)		507,598 (85.4)				7419 (72.1)		20,312 (74.8)		0.086		37,007 (81.4)		92,180 (82.6)			
	Not severe conditions	38,742 (9.6)	135,022 (9.8)			21,005 (10.4)		64,581 (10.9)				1631 (15.8)		4296 (15.8)				5258 (11.6)		13,052 (11.7)			
	Severe conditions	13,308 (3.3)	39,426 (2.9)			8580 (4.3)		22,540 (3.8)				1241 (12.1)		2561 (9.4)				3218 (7.1)		6369 (5.7)			

^a^SMD: significant mean difference.

^b^CCI: Charlson Comorbidity Index.

### Health Care Use

We compared the number of health care uses associated with the diagnosis of each chronic disease. During the post–COVID-19 pandemic period, health care use decreased; however, the decrease in the telemedicine group was less than that in the control group for all patients (mean difference: –0.06 vs –0.28, *P*<.001 for the hypertension group; –0.06 vs –0.37, *P*<.001 for the DM group; and –0.56 vs –0.79, *P*=.001 for the common mental disorder group), except COPD (–0.79 vs –0.80; *P*=.40). The average number of telemedicine visits in 1 year after the telemedicine policy was implemented was 1.95 (SD 1.86) for the hypertension group, 1.89 (SD 1.76) for the DM group, 1.66 (SD 1.62) for the COPD group, and 2.52 (SD 3.74) for the common mental disorder group (Table 2).

**Table 2:** The number of health care uses in the telemedicine group and control group 1 year before and 1 year after the telemedicine policy was implemented.

Disease groups			The number of health care uses for 1 year, mean (SD)			Difference^a^		*P* value
			Preintervention period^b^		Postintervention period^c^			
**Hypertension**								
	Telemedicine (only telemedicine)	8.74 (5.31)		8.68 (5.30)^d^ 1.95 (1.86)^e^		–0.06	—^f^	
	Control	8.45 (5.45)		8.17 (5.40)		–0.28	—	
	Difference-in-difference	—		—		0.20	<.001	
**Diabetes mellitus**								
	Telemedicine (only telemedicine)	9.89 (6.85)		9.83 (6.71)^d^ 1.89 (1.76)^e^		–0.06	—	
	Control	9.62 (7.20)		9.25 (7.19)		–0.37	—	
	Difference-in-difference	—		—		0.27	<.001	
**Chronic obstructive pulmonary disease**								
	Telemedicine (only telemedicine)	6.43 (6.37)		5.63 (6.10)^d^ 1.66 (1.62)^e^		–0.79	—	
	Control	6.31 (5.84)		5.46 (5.45)		–0.85	—	
	Difference-in-difference	—		—		0.10	.40	
**Common mental disorders**								
	Telemedicine (only telemedicine)	9.36 (7.83)		8.80 (7.93)^d^ 2.52 (3.74)^e^		–0.56	—	
	Control	8.72 (7.29)		7.93 (7.35)		–0.79	—	
	Difference-in-difference	—		—		0.22	.001	

^a^Difference: values were calculated by subtracting numbers of the postintervention period from the preintervention period.

^b^Preintervention period: before 1 year of telemedicine introduction.

^c^Postintervention period: after 1 year of telemedicine introduction.

^d^The total health care usage count for the telemedicine group,

^e^The count specifically for telemedicine usage.

^f^Not applicable.

### MPR Changes

The effect of telemedicine on medication prescriptions, in terms of the MPR and telemedicine policy, resulted in an overall increase in the MPR for both patients with hypertension and DM. For patients with hypertension, the MPR increased in both the telemedicine group (83.8% vs 87%) and the control group (83.9% vs 86.6%); however, the increase was more significant in the telemedicine group, with a 0.5% DID (*P*<.001). For patients with DM, similar to patients with hypertension, the MPR increased in both the telemedicine group (83.9% vs 87.3%) and the control group (84.2% vs 87.4%), and the difference was greater in the telemedicine group (0.2% DID; *P*=.009). Moreover, the improvement in the MPR for the COPD and common mental disorder groups was not significant. For patients with COPD, the MPR increased after the policy was implemented in both the telemedicine group (46.4% vs 52.5%) and the control group (43.9% vs 50%); however, the effects of the telemedicine policy were not significant (–0.1% DID; *P*=.94). Similarly, for patients with common mental disorders, the MPR increased in the telemedicine group (55.3% vs 64.2%) and control group (52.90% vs 61.80%) after the policy was implemented; however, the telemedicine policy effects were not significant (–0.1% DID; *P*=.78; Table 3 and Multimedia Appendices 3-6). The monthly changes in MPR before and after policy implementation are presented in Multimedia Appendix 7.

**Table 3:** Differences in the medication possession ratio among patients with chronic diseases.

Disease groups			Medication possession ratio (%)					Difference^a^			*P* value^b^
			Preintervention period^c^		Postintervention period^d^						
**Hypertension**											
	Telemedicine	83.85%		87%		3.15%			—^e^		
	Control	83.93%		86.62%		2.69%			—		
	Difference-in-differences	—		—		0.5%			<.001		
**Diabetes mellitus**											
	Telemedicine	83.87%		87.31%		3.44%			—		
	Control	84.22%		87.42%		3.2%			—		
	Difference-in-differences	—		—		0.2%			.009		
**Chronic obstructive pulmonary disease**											
	Telemedicine	46.42%		52.47%		6.05%			—		
	Control	43.93%		50.01%		6.08%			—		
	Difference-in-differences	—		—		–0.1%			.94		
**Common mental disorders**											
	Telemedicine	55.31%		64.16%		8.85%			—		
	Control	52.89%		61.82%		8.93%			—		
	Difference-in-differences	—		—		–0.1%			.72		

^a^Difference: values were calculated by subtracting numbers of the postintervention period from the preintervention period.

^b^*P* values were calculated by multiple regression model equation with age, sex, residence, Charlson Comorbidity Index, and disability adjustment.

^c^Preintervention period: before 1 year of telemedicine introduction.

^d^Postintervention period: after 1 year of telemedicine introduction.

^e^Not applicable.

### Admission Rates to EDs, GWs, and ICUs

The implementation of the telemedicine policy was associated with a decrease in hospitalization rates for patients with hypertension and DM, while its impact on patients with COPD and common mental disorders was not statistically significant (all *P*>.05). For patients with hypertension, there were decreases in GW admissions (–0.1%; *P*<.001), ED visits (–0.2%; *P*<.001), and ICU admissions (–0.1%; *P*<.001). In patients with DM, there were reductions in GW (–0.1%; *P*=.02) and ED admissions (–0.1%; *P*=.047); however, the influence of telemedicine on ICU admissions was not significant (*P*=.09; Table 4).

Analysis of hospital admission rates within 1 month after health care use in each disease group revealed that patients with DM had greater GW (0.32% vs 0.17%; *P*<.001), ED (0.16% vs 0.11%; *P*<.001), and ICU (0.06% vs 0.05%; *P*=.001) admission rates after in-person visits than after telemedicine visits. For patients with hypertension, in-person visits resulted in higher GW (0.13% vs 0.09%; *P*<.001) and ICU admission rates (0.11% vs 0.1%; *P*<.001), but admissions after ED visits (0.05% vs 0.03%; *P*=.28) were not significantly different from those after telemedicine visits. For patients with COPD, there was no significant difference in admission rates within 1 month between telemedicine and in-person visits (all *P*>.05). For patients with common mental disorders, telemedicine was associated with lower GW admission rates (0.12% vs 0.21%; *P*<.001) only, with no significant differences in other admission rates (all *P*>.05; Table 5).

**Table 4:** The proportions of patients admitted to general wards, emergency departments, and intensive care units in the telemedicine group and the control group 1 year before and 1 year after the telemedicine policy was implemented.

Admission type		General ward					Emergency department					Intensive care unit			
		Preintervention period^a^	Postintervention period^b^	Difference^c^	*P* value	Preintervention period^a^		Postintervention period^b^	Difference^c^	*P* value	Preintervention period^a^		Postintervention period^b^	Difference^c^	*P* value
**Hypertension, (%)**															
	Telemedicine	1.14	1.13	–0.01	—^d^	1.06		1.13	0.07	—	0.28		0.38	0.10	—
	Control	1.05	1.19	0.14	—	0.89		1.13	0.24	—	0.26		0.42	0.16	—
	Difference-in-differences	—	—	–0.10	<.001	—		—	–0.17	<.001	—		—	–0.06	<.001
**Diabetes mellitus (%)**															
	Telemedicine	2.55	2.07	–0.47	—	1.43		1.35	–0.08	—	0.39		0.44	0.05	—
	Control	2.45	2.11	–0.35	—	1.30		1.30	0.00	—	0.38		0.46	0.09	—
	Difference-in-differences	—	—	–0.13	.02	—		—	–0.08	.047	—		—	–0.04	.09
**Chronic obstructive pulmonary disease (%)**															
	Telemedicine	10.63	6.31	–4.32	—	8.80		5.33	–3.47	—	0.59		0.86	0.26	—
	Control	10.01	5.64	–4.37	—	7.62		4.64	–2.98	—	0.72		0.81	0.09	—
	Difference-in-differences	—	—	0.04	.92	—		—	–0.49	.22	—		—	0.17	.23
**Common mental disorders (%)**															
	Telemedicine	1.78	1.08	–0.70	—	0.51		0.31	–0.20	—	0.01		0.00	0.00	—
	Control	1.45	0.89	–0.57	—	0.32		0.17	–0.15	—	0.00		0.01	0.00	—
	Difference-in-differences	—	—	–0.14	.11	—		—	–0.05	.29	—		—	–0.01	.18

^a^Preintervention period: before 1 year of telemedicine introduction.

^b^Postintervention period: after 1 year of telemedicine introduction.

^c^Difference: values were calculated by subtracting numbers of the postintervention period from the preintervention period.

^d^Not applicable.

**Table 5:** The number of events involving admission to general wards, emergency departments, and intensive care units within 1 month of each telemedicine use and in-person visit.

Admission type	Hypertension			Diabetes mellitus				Chronic obstructive pulmonary disease				Common mental disorders		
	Telemed-icine (n= 415,739)	In-person (n= 14,267,444)	*P* value	Telemed-icine (n= 217,426)	In-person (n= 7,516,533)	*P* value	Telemed-icine (n= 7,216)		In-person (n= 212,302)	*P* value	Telemed-icine (n= 36,005)		In-person (n= 1,248,602)	*P* value
General ward admission, n (%)	363 (0.09)	19,056 (0.13)	<.001	377 (0.17)	23,812 (0.32)	<.001	59 (0.82)		2180 (1.03)	.09	45 (0.12)		2651 (0.21)	<.001
Emergency department, n (%)	420 (0.03)	15,230 (0.05)	.28	242 (0.11)	12,232 (0.16)	<.001	54 (0.75)		1531 (0.72)	.84	16 (0.04)		605 (0.05)	.83
Intensive care unit, n (%)	129 (0.10)	6584 (0.11)	<.001	98 (0.05)	4743 (0.06)	.001	14 (0.19)		296 (0.14)	.29	1 (0)		21 (0)	1

## Discussion

### Principal Findings

This study examined the impact of a telemedicine policy on health care use, medication prescription adherence, and admission rates in the early stages among certain chronic disease groups using a DID analysis. In addition, this research highlights the outcomes and early effects of a national telemedicine policy that was implemented during the COVID-19 pandemic; notably, telemedicine consultations were conducted amid the outbreak.

In terms of the patient’s demographics, older patients, particularly those in their 70s, and those living in rural areas generally showed lower telemedicine use. This finding is consistent with previous reports, which attribute the trend to lower digital literacy among older age groups, limited technological infrastructure, or a preference for in-person visits in rural areas [41,42].

We found that patients diagnosed with hypertension or DM who used telemedicine had an increased MPR without increasing rates of admission to GWs, EDs, or ICUs during the first year after the policy was implemented. A major reason for these results could be that telemedicine helped improve adherence to antihypertensive and antidiabetic medications, which are critical factors in managing these disease groups [36,43,44]. We suggest that telemedicine can mitigate the decrease in health care use during lockdown periods and increase prescription adherence, ultimately enhancing the MPR and leading to lower admission rates, thereby improving disease management. When assessing the risk of adverse events associated with telemedicine use, telemedicine visits did not result in increased admission rates compared with in-person visits. Some previous studies support our findings, indicating that telemedicine improves engagement in continuous care and has a positive effect on clinical outcomes, such as lowering hemoglobin A_1c_ levels and managing BP [45-47]. However, these trends were not observed for patients with COPD or common mental disorders.

Telemedicine for patients with COPD has shown potential in various areas, such as remote education, disease monitoring, cost-effectiveness, and improved access to health care, with studies indicating positive outcomes in rehabilitation enhancement [48-50]. However, despite previous reports, the effects of telemedicine on reducing hospital admissions or exacerbations and improving medication adherence remain uncertain, which is consistent with the findings of our study [51,52]. We infer that the relatively older age of patients with COPD than of patients with other chronic diseases may be linked to these outcomes. Telemedicine adoption often requires familiarization with new platforms, such as mobile apps, which can be challenging for older individuals with limited digital literacy [53,54]. Furthermore, low medication adherence, often due to complex regimens, may account for the unclear impact of telemedicine on medication adherence observed in our study [55].

In our study, the patients in the common mental disorder group, similar to those in the COPD group, displayed a low MPR, and telemedicine did not improve the MPR under these conditions. Telemedicine interventions, such as telephone cognitive behavioral therapy, have been widely adopted in psychiatry. The exclusion of certain types of telemedicine treatment might have limited the observed effectiveness in our study. Nonetheless, during the COVID-19 pandemic, there was a marked preference among patients and health care providers for continuing the use of telemedicine, reflecting its potential benefits in terms of convenience and access to care, although the impact on the MPR and admission rates remained unclear [56-58]. Furthermore, a few studies have indicated that telemedicine, particularly psychotherapy in psychiatry, can broaden treatment possibilities for patients with mood and anxiety disorders, offering significant benefits for elderly patients [59].

In future studies, the long-term effects of telemedicine will need to be evaluated to establish it as a permanent and sustainable national solution. Moreover, assessing the effectiveness of telemedicine requires exploring nonmedication-related outcomes such as clinical laboratory test results, symptom severity, and mortality. In addition, the influence of various telemedicine modalities on disease characteristics should be explored. Identifying the optimal telemedicine approach for the most suitable target populations should be a focus of future research.

### Limitations

First, the data included a 5-fold control group matched to the telemedicine group based on age and sex for all telemedicine users, although not for the entire population, due to NHIS policy restrictions. As a result, variables such as socioeconomic status and comorbidities were not directly matched. While we incorporated fixed effects, such as region and the CCI, in the DID analysis to account for some of these factors, residual confounding may still remain.

Second, although we did not apply survey weights during the analysis, the age and sex distributions in our data were similar to national disease demographics across 4 disease groups [60,61]. Finally, our study focuses on a specific period during the COVID-19 pandemic, when telemedicine adoption surged due to policy changes. Therefore, applying these findings to nonpandemic contexts or different health care settings should be done with caution.

### Conclusion

The temporary telemedicine policy effectively increased medication adherence and reduced admission rates for patients with hypertension and DM. However, the effectiveness of the policy was limited for patients with COPD and common mental disorders when analyzed using national claims data. Future studies are needed to demonstrate the long-term effects of telemedicine policies, considering various outcome measures and disease characteristics.

## Appendix

Multimedia Appendix 1 Operational definition of each chronic disease.

Multimedia Appendix 2 Sociodemographic variables.

Multimedia Appendix 3 Difference-in-differences analysis result for multilinear regression model about medication possession ratio: hypertension.

Multimedia Appendix 4 Difference-in-differences analysis results for multilinear regression model about medication possession ratio: diabetes mellitus.

Multimedia Appendix 5 Difference-in-differences analysis results for multilinear regression model about medication possession ratio: chronic obstructive pulmonary disease.

Multimedia Appendix 6 Difference-in-differences analysis results for multilinear regression model about medication possession ratio: common mental disorders.

Multimedia Appendix 7 Monthly plot of the medication possession ratio trend before and after policy implementation over 12 months.

## Abbreviations

BP: blood pressure
CCI: Charlson Comorbidity Index
COPD: chronic obstructive pulmonary disease
DID: difference in differences
DM: diabetes mellitus
ED: emergency department
GW: general ward
HTN: hypertension
ICU: intensive care unit
KCD-7: Korean Classification of Diseases, 7th Revision
MPR: medication possession ratio
NHIS: National Health Insurance Service
STROBE: Strengthening the Reporting of Observational Studies in Epidemiology

## Data Availability

The datasets analyzed during this study are not publicly available due to policy of the National Health Insurance Service in South Korea but are available from the corresponding author on reasonable request.
