# Supplementary material for: Temporary Telemedicine Policy and Chronic Disease Management in South Korea: Retrospective Analysis Using National Claims Data
Source: JMIR Public Health Surveill. 2024 Nov 20;10:e59138. doi: 10.2196/59138 (PMC11618008; doi:10.2196/59138)
Supplement: Multimedia Appendix 1 [file publichealth_v10i1e59138_app1.docx]

**Multimedia Appendix 1.**

| **Chronic disease** | **Inclusion diagnosis based on ICD-10 code** | **Prescribed drug classes** |
| --- | --- | --- |
| Hypertension | I10(Essential (primary) hypertension), I11(Hypertensive heart disease), I12(Hypertensive renal disease), I13(Hypertensive heart and renal disease), I15(Secondary hypertension) | 212(Antiarrhythmic agents), 213(Diuretic), 214(Antihypertensives), 217(Vasodilator), 219(Other circulatory agents) |
| Diabetes mellitus | E10(Type 1 diabetes mellitus), E11(Type 2 diabetes mellitus), E12(Malnutrition-related diabetes mellitus), E13(Other specified diabetes mellitus), E14(Unspecified diabetes mellitus) | 396(Antidiabetics) |
| Chronic obstructive pulmonary disease | J43(Emphysema), J43.1(Panlobular emphysema), J43.2(Centrilobular emphysema), J43.8(Other emphysema), J43.9(Emphysema, unspecified), J44(Other chronic obstructive pulmonary disease), J44.0(Chronic obstructive pulmonary disease with acute lower respiratory infection), J44.1(Chronic obstructive pulmonary disease with acute exacerbation, unspecified), J44.8(Other specified chronic obstructive pulmonary disease), J44.9(Chronic obstructive pulmonary disease, unspecified) | 222(Cough and expectorants medicine), 229(Other drugs acting on respiratory organ) |
| Common mental disorders | F20(Schizophrenia), F21(Schizotypaldisorder), F22(Persistent delusional disorders), F23(Acute and transient psychotic disorders), F24(Induced delusional disorder), F25(Schizoaffective disorders), F28(Other nonorganic psychotic disorders), F29(Unspecified nonorganic psychosis), F31(Bipolar affective disorder), F32(Depressive episode), F33(Recurrent depressive disorder), F41(Other anxiety disorders) | 112(Hypnotic and sedatives), 113(Antiepileptics), 115(Stimulant), 117(Tranquilizer), |
