# Supplementary material for: Temporary Telemedicine Policy and Chronic Disease Management in South Korea: Retrospective Analysis Using National Claims Data
Source: JMIR Public Health Surveill. 2024 Nov 20;10:e59138. doi: 10.2196/59138 (PMC11618008; doi:10.2196/59138)
Supplement: Multimedia Appendix 2 [file publichealth_v10i1e59138_app2.docx]

**Multimedia Appendix 2**.

|  | **Condition** | |
| --- | --- | --- |
| Age | 18-39/ 40-49/ 50-59/ 60-69/ 70-79/ 80+ | |
| Gender | Female/ Male | |
| Charlson Comorbidity Index (CCI) | - Classification using the diagnosis records of major diseases of target patients from the year before the policy implementation. - Age and 19 comorbidities are scored and classified into scores from 0 to 3 - Score: 0, 1, 2, 3+ | |
| Residence | - Based on the National Statistical Office's population survey of April 2023. Metropolitan cities defined as cities with more than 1 million people. | |
|  | Metropolis | Seoul City, Busan City, Incheon City, Daegu City, Daejeon City, Gwangju City, Suwon City, Ulsan City, Go-yang City, Gyeonggi-do, Yong-in City, Gyeongsangnam-do, Changwon, Gyeongsangnam-do |
|  | City | A city of South Korea that is not included in a metropolis (i.e. Pohang City, Gyeongsangbuk-do, Jeju Special Self-Governing Province) |
|  | Rural | the rest of the country, except for large and urban areas |
| Type of disability | Physical disability | Applicable to all types of disabilities except mental disorders |
|  | Psychiatric disability | Mental disabilities include 06: intellectually disabled, 07: autistic disabled, and 08: mentally disabled. |
| Degree of disability | Mild disability | The disability level is 4 to 6, and the disability level code (CD_VL_NM1) is code 20 (mild). |
|  | Severe disability | The disability level corresponds to grades 1 to 3, and the disability level code (CD_VL_NM1) is code 10 (severe). |
