# Supplementary material for: Temporary Telemedicine Policy and Chronic Disease Management in South Korea: Retrospective Analysis Using National Claims Data
Source: JMIR Public Health Surveill. 2024 Nov 20;10:e59138. doi: 10.2196/59138 (PMC11618008; doi:10.2196/59138)
Supplement: Multimedia Appendix 3 [file publichealth_v10i1e59138_app3.docx]

**Multimedia Appendix 3.**

|  | **Estimate** | **SE**^a^ | ***P* value** |
| --- | --- | --- | --- |
| **Intercept** | 0.83 | 0.0004 | <.001 |
| **Treatment after** | 0.005 | 0.001 | <.001 |
| **Age (18-59)** | | | |
| 60-69 | 0.037 | 0.0003 | <.001 |
| 70-79 | 0.044 | 0.0003 | <.001 |
| 80- | 0.021 | 0.0004 | <.001 |
| **Gender (female)** | | | |
| Male | –0.003 | 0.0003 | <.001 |
| **Residence (metropolis)** | | | |
| City | –0.011 | 0.0003 | <.001 |
| Rural | –0.025 | 0.0004 | <.001 |
| **Charlson comorbidity index (0)** | | | |
| 1 | –0.004 | 0.0003 | <.001 |
| 2 | –0.012 | 0.0004 | <.001 |
| 3+ | –0.036 | 0.0004 | <.001 |
| **The type of disability (normal)** | | | |
| Physical disability | –0.013 | 0.001 | <.001 |
| Psychiatric disability | –0.02 | 0.002 | <.001 |
| **The degree of disability (normal)** | | | |
| Not severe conditions | 0.006 | 0.001 | <.001 |
| Severe conditions |  |  |  |

^a^S.E.: Standard Error.
