# Supplementary material for: Temporary Telemedicine Policy and Chronic Disease Management in South Korea: Retrospective Analysis Using National Claims Data
Source: JMIR Public Health Surveill. 2024 Nov 20;10:e59138. doi: 10.2196/59138 (PMC11618008; doi:10.2196/59138)
Supplement: Multimedia Appendix 4 [file publichealth_v10i1e59138_app4.docx]

**Multimedia Appendix 4.**

|  | **Estimate** | **SE**^a^ | ***P* value** |
| --- | --- | --- | --- |
| **Intercept** | 0.815 | 0.009 | <.001 |
| **Treatment after** | 0.002 | 0.001 | 0.009 |
| **Age (18-59)** |  |  |  |
| 60-69 | 0.049 | 0.001 | <.001 |
| 70-79 | 0.053 | 0.001 | <.001 |
| 80- | 0.031 | 0.001 | <.001 |
| **Gender (female)** |  |  |  |
| Male | –0.003 | 0.0004 | <.001 |
| **Residence (metropolis)** |  |  |  |
| City | –0.012 | 0.0004 | <.001 |
| Rural | –0.025 | 0.001 | <.001 |
| **Charlson comorbidity index (0)** |  |  |  |
| 1 | 0.004 | 0.009 | 0.672 |
| 2 | 0.002 | 0.009 | 0.805 |
| 3+ | 0.003 | 0.009 | 0.749 |
| **The type of disability (normal)** |  |  |  |
| Physical disability | –0.024 | 0.001 | <.001 |
| Psychiatric disability | 0.002 | 0.003 | 0.488 |
| **The degree of disability (normal)** |  |  |  |
| Not severe conditions | 0.019 | 0.001 | <.001 |
| Severe conditions |  |  |  |

^a^S.E.: Standard Error.
