# Supplementary material for: Temporary Telemedicine Policy and Chronic Disease Management in South Korea: Retrospective Analysis Using National Claims Data
Source: JMIR Public Health Surveill. 2024 Nov 20;10:e59138. doi: 10.2196/59138 (PMC11618008; doi:10.2196/59138)
Supplement: Multimedia Appendix 5 [file publichealth_v10i1e59138_app5.docx]

**Multimedia Appendix 5.**

|  | **Estimate** | **SE**^a^ | ***P* value** |
| --- | --- | --- | --- |
| **Intercept** | 0.282 | 0.007 | <.001 |
| **Treatment after** | –0.001 | 0.008 | 0.941 |
| **Age (18-59)** |  |  |  |
| 60-69 | 0.074 | 0.007 | <.001 |
| 70-79 | 0.118 | 0.007 | <.001 |
| 80- | 0.154 | 0.007 | <.001 |
| **Gender (female)** |  |  |  |
| Male | 0.049 | 0.004 | <.001 |
| **Residence (metropolis)** |  |  |  |
| City | 0.024 | 0.004 | <.001 |
| Rural | 0.024 | 0.005 | <.001 |
| **Charlson comorbidity index (0)** |  |  |  |
| 1 |  |  |  |
| 2 | –0.005 | 0.004 | 0.227 |
| 3+ | –0.047 | 0.004 | <.001 |
| **The type of disability (normal)** |  |  |  |
| Physical disability | 0.159 | 0.006 | <.001 |
| Psychiatric disability | 0.103 | 0.029 | <.001 |
| **The degree of disability (normal)** |  |  |  |
| Not severe conditions | –0.134 | 0.007 | 0.941 |
| Severe conditions |  |  |  |

^a^S.E.: Standard Error.
