# Supplementary material for: Temporary Telemedicine Policy and Chronic Disease Management in South Korea: Retrospective Analysis Using National Claims Data
Source: JMIR Public Health Surveill. 2024 Nov 20;10:e59138. doi: 10.2196/59138 (PMC11618008; doi:10.2196/59138)
Supplement: Multimedia Appendix 6 [file publichealth_v10i1e59138_app6.docx]

**Multimedia Appendix 6.**

|  | **Estimate** | **SE**^a^ | ***P* value** |
| --- | --- | --- | --- |
| **Intercept** | 0.530 | 0.002 | <.001 |
| **Treatment after** | –0.001 | 0.003 | 0.72 |
| **Age (18-59)** |  |  |  |
| 60-69 | –0.004 | 0.002 | 0.063 |
| 70-79 | 0.004 | 0.002 | 0.061 |
| 80- | 0.044 | 0.002 | <.001 |
| **Gender (female)** | | | |
| Male | 0.004 | 0.002 | 0.007 |
| **Residence (metropolis)** | | | |
| City | –0.001 | 0.002 | 0.539 |
| Rural | –0.031 | 0.002 | <.001 |
| **Charlson comorbidity index (0)** | | | |
| 1 | –0.011 | 0.002 | <.001 |
| 2 | –0.025 | 0.002 | <.001 |
| 3+ | –0.044 | 0.002 | <.001 |
| **The type of disability (normal)** | | | |
| Physical disability | 0.042 | 0.004 | <.001 |
| Psychiatric disability | 0.220 | 0.004 | <.001 |
| **The degree of disability (normal)** | | | |
| Not severe conditions | –0.030 | 0.004 | <.001 |
| Severe conditions |  |  |  |

^a^S.E.: Standard Error.
