# Supplementary material for: Temporary Telemedicine Policy and Chronic Disease Management in South Korea: Retrospective Analysis Using National Claims Data
Source: JMIR Public Health Surveill. 2024 Nov 20;10:e59138. doi: 10.2196/59138 (PMC11618008; doi:10.2196/59138)
Supplement: Multimedia Appendix 7 [file publichealth_v10i1e59138_app7.docx]

**Multimedia Appendix 7**

**Monthly plot of the medication possession ratio trend before and after policy implementation over 12 months.**

1. **Control group**

**
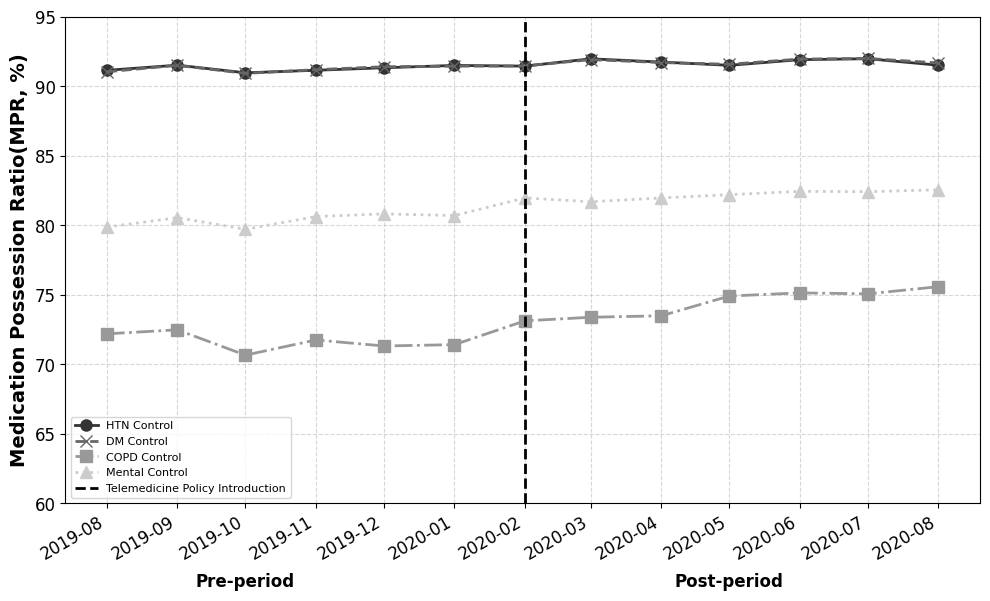
**

1. **Telemedicine group**

**
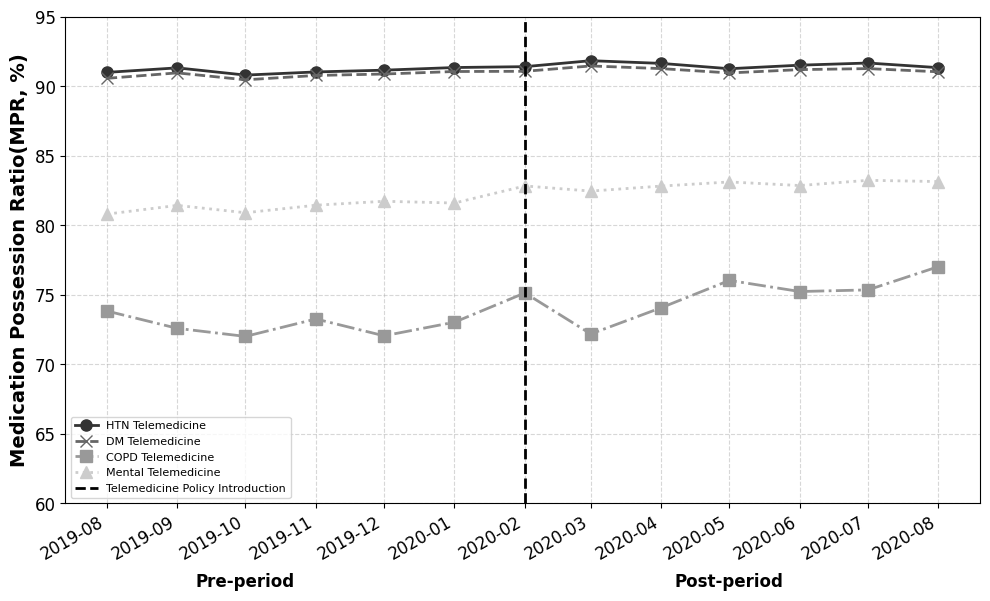
**

*The MPR calculation includes both outpatient and inpatient medication prescriptions.
